# Supplementary material for: Resource depletion through primate stone technology
Source: eLife. 2017 Sep 8;6:e23647. doi: 10.7554/eLife.23647 (PMC5590808; doi:10.7554/eLife.23647)
Supplement: Source data 2. — The size and maturation stages of the main prey species harvested by tool using macaques on Koram and NomSao Island, Thailand. [file elife-23647-data2.docx]

Source data 4:

Maturation stages of gastropods between Koram and NomSao Island in Thailand

| Location | Size | *M. Labio* | | *P.sulcatus* | *C. bifasciatus* |
| --- | --- | --- | --- | --- | --- |
| NomSao | 1 | +  0  0  0 | 0  0  0 | | ++  0  0  0  0  0  + |
|  | 2 | +++  +++  +++  +++ | +  +  0  +  0  0  0  0 | | +++  +++  +++  +++ |
|  | 3 |  | ++  +++  +++  +++  +++ | |  |
|  | 4 |  | +++  +++  +++  +++  +++  +++  +++ | |  |
| Koram | 1 | ++  ++  0  +  0 | 0  0  ++  0  0  0 | | 0  0  0  +  +  0  0  0  0 |
|  | 2 |  | 0  0  +  +  ++ | | ++  ++  +++  +++ |
|  | 3 |  | +++  +++  +++  +++  +++  +++ | |  |

Criteria on scoring the shell maturity with the size of ovary/testis:

+++ fully mature: testis/ovary fully occupy (>80%) the upper whorl

++ mature: testis/ovary occupy (>50%) of the upper whorl

+ sub-adult: testis/ovary occupy less than 50% of the upper whorl

0 immature: no traces of testis or ovary
